# Radiotherapy for Lung Metastases in a Patient With Ewing Sarcoma

**DOI:** 10.1080/13577149878000

**Published:** 1998-12

**Authors:** A. Kegye, A. Naszály

**Affiliations:** Center of Oncoradiology Uzsoki Hospital Uzsoki u. 29 Budapest H-1145 Hungary

## Abstract

*Purpose.* To assess the benefit of therapy for multiple lung metastases in a young female patient previously treated with operation, local radiotherapy and chemotherapy.

*Patient:* Lung metastases occurred in a young female patient 13 months after finishing combined treatment of a Ewing sarcoma of the left eleventh rib. Primary treatment had included surgical removal, 51 Gy local radiotherapy and chemotherapy.

*Method.* 20 Gy total dose was applied to the lungs of both sides in two courses with an additional 15 Gy to the mediastinum.

*Results and Discussion.* Complete radiological regression was achieved at the end of therapy which was maintained during the follow-up period of 16 months.


*Sarcoma* (1998) **2**, 209–213

CASE REPORT

### Radiotherapy for lung metastases in a patient with Ewing sarcoma

A. KEGYE & A. NASZÁLY

*Center of Oncoradiology, Uzsoki Hospital, H-1145 Budapest, Uzsoki u. 29, Hungary*

**Abstract**

*Purpose.* To assess the benefit of therapy for multiple lung metastases in a young female patient previously treated with operation, local radiotherapy and chemotherapy.

*Patient*: Lung metastases occurred in a young female patient 13 months after finishing combined treatment of a Ewing sarcoma of the left eleventh rib. Primary treatment had included surgical removal, 51 Gy local radiotherapy and chemotherapy.

*Method.* 20 Gy total dose was applied to the lungs of both sides in two courses with an additional 15 Gy to the mediastinum.

*Results and Discussion.* Complete radiological regression was achieved at the end of therapy which was maintained during the follow-up period of 16 months.

**Key words**: *Ewing sarcoma, lung metastases, palliative radiotherapy.*

### Introduction

Ewing sarcoma is the second most frequent bone tumour among children and young adults. Comprises 10–15% of all bone tumors. It rarely occurs under the age of 5 or above the age of 30. The male to female ratio is 1.5:1.

Ewing sarcoma arises intramedullary, breaks through the cortex and extend as a soft tissue mass. Virtually any bone can be the site of the primary lesion, but the lower half of the body is much more frequently involved than the upper half. The frequency of rib involvement follows that of the femur, the pelvic bones, the fibula, the humerus and the tibia. It has a moderately bad prognosis.[1–3] Distant metastases are frequent to the lungs and other bones. Metastatic involvement of lymph nodes occurs in less than 10% of cases. Increasing pain, swelling and fever are the leading clinical symptoms.

Plain X-ray with bone and bone marrow scintigraphy demonstrate primary or secondary skeletal involvement. Computed tomography (CT) and MRI are informative for the judgement of periosteal and the intramedullary extension.[2]

### Diagnosis

A 29-year-old female patient presented with increasing back pain on the left side, effort dyspnoea and fever. Chest X-ray showed a palm-sized supraphrenic infiltration on the left with minimal pleural effusion. Laboratory parameters were normal except for a raised sedimentation rate and leucocytosis. Pneumonia was suspected and antibiotic treatment started but dyspnoea and the level of the pleural effusion increased. Cytology of the pleural fluid suggested inflammation but further X-ray showed extensive confluent rarefaction of the XIth rib with bone destruction. Whole body bone and bone marrow scan showed no changes elsewhere.

### Primary treatment

After resection of the involved rib, histopathological examination (including immuno-histochemistry) gave the diagnosis of Ewing sarcoma. Postoperative radiochemotherapy was commenced in June 1994. The chemotherapy comprised 6 courses of VIP (200 mg Vepeside, 800 mg Ifosfamide, 60 mg Cisplatin, with Uromitexan rescue). Radiotherapy was delivered after the first course of chemotherapy (51 Gy locally with a high-energy electron beam). The first course of chemotherapy had to be interrupted because of dysuria. Colony stimulating factor was necessary because of leukopenia after further courses.

Correspondence to: Adrienne Kegye, Center of Oncoradiology, Uzsoki Hospital, Budapest, Uzsoki u. 29, Hungary H-1145. Fax: 36 1 251 7577.

1357-714X/98/030209-05 $9.00 © 1998 Carfax Publishing Ltd

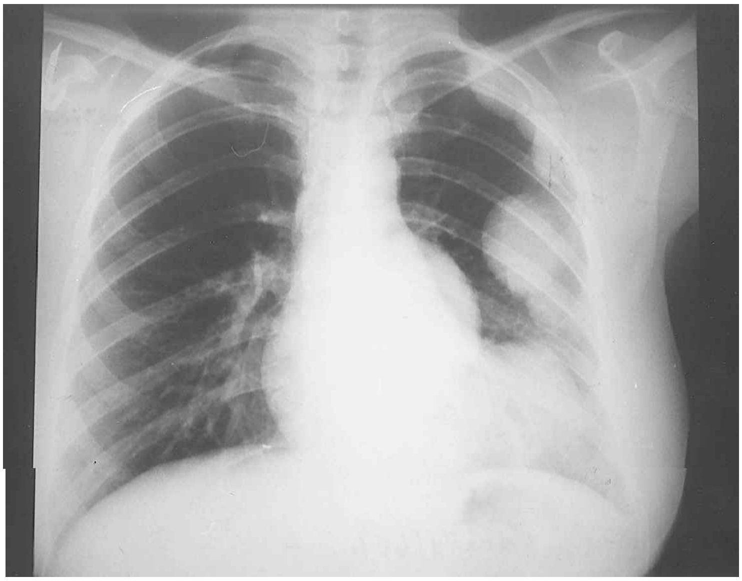

**Fig. 1.** *Plain X-ray before pulmonary irradiation.*

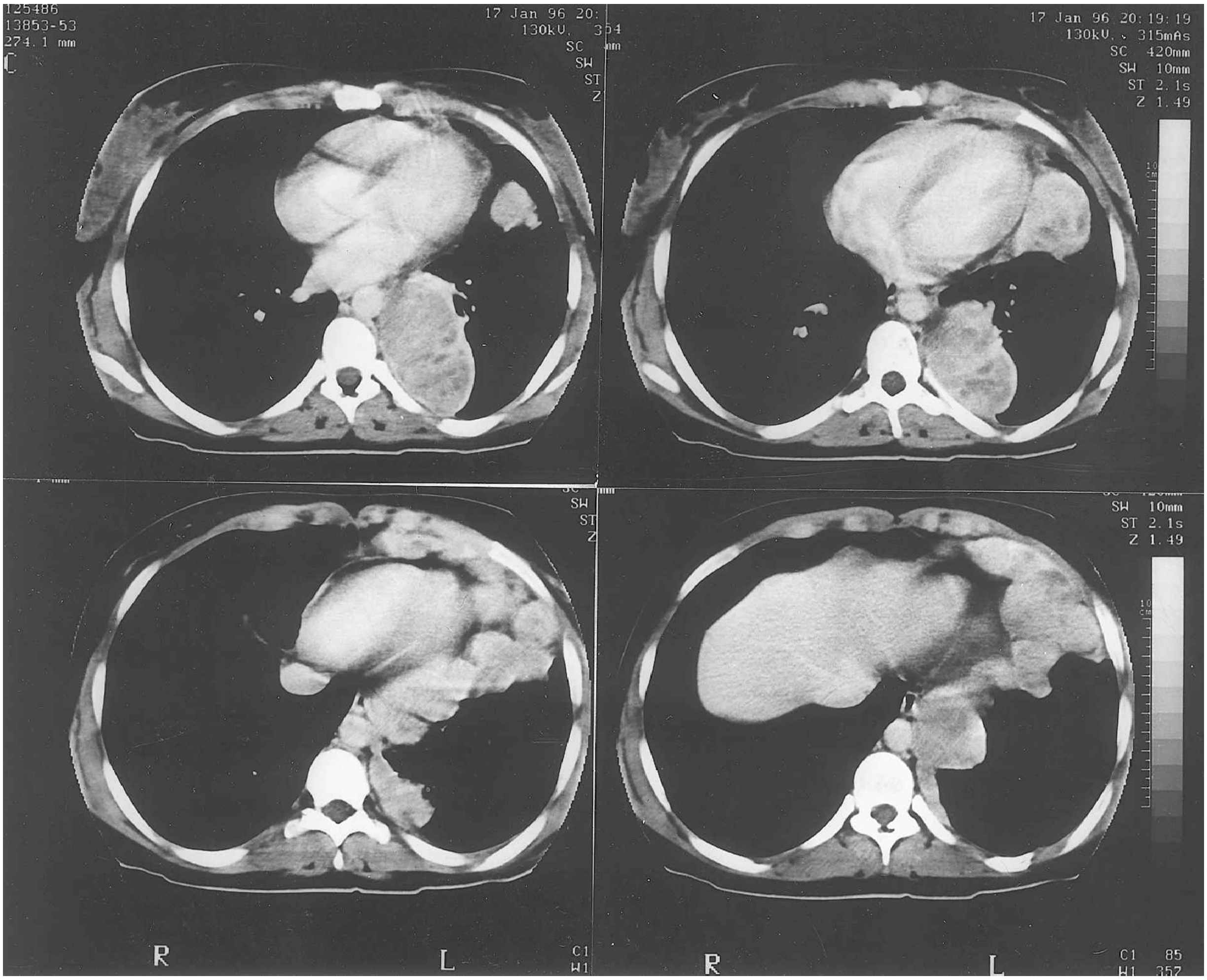

**Fig. 2.** *CT scan before pulmonary irradiation.*

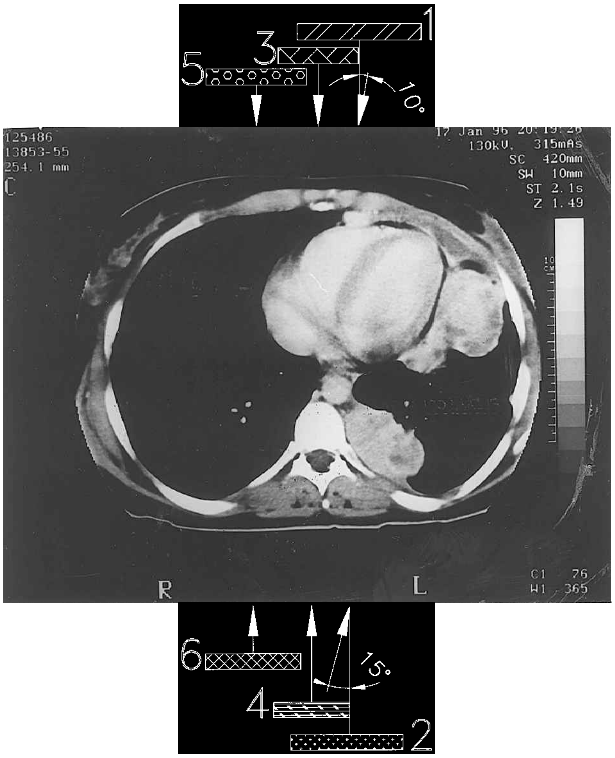

**Fig. 3.** *Treatment scheme: first phase: portals 1 and 2, left hemithorax; second phase: portals 3 and 4, mediastinum; and third phase: portals 5 and 6, right hemithorax.*

### Treatment of the pulmonary metastases

She remained symptom free for 13 months, with complete remission documented by CT. After the onset of cough pulmonary metastases were shown on thoracic X-ray and CT. Multiple rounded opacities were found: paracardic anteriorly 7 cm, retrocardiac 8 cm, lateral to the left hilus 6 cm, in the left apex along the thoracic wall on the pleura 8 cm, and above the arch of aorta along the mediastinal pleura 3 cm in diameter. The right lung showed no abnormality (Figs 1 and 2).

Whole-thorax irradiation was performed in three phases. An effective palliation was the aim of therapy in this case, so the left hemithorax was treated first. After experiencing the very rapid regression of the multiple lesions in this site, treatment was extended to the neighbouring structures at risk, i.e. mediastinal and prophylactic right hemithorax irradiation was performed in the following two treatment phases. 20 Gy total mid-plane dose was applied to the involved left hemithorax from a telecobalt unit with slightly angled anterior and posterior portals using 1.5 Gy fractions. The anterior field was tilted 10 degrees laterally and the posterior field 15 degrees medially. This slight tilting enabled the paravertebral entering of the medial edge of the posterior portal with respect to the better protection of the spinal cord after previous chemotherapy. Three weeks later plain X-ray showed complete remission of the metastases on the left side.

Complementary mediastinal irradiation was given after a six weeks interruption, with 15 Gy mid-plane were applied via central-symmetrically positioned, opposed portals in 1.5 Gy fractions.

Prophylactic irradiation of the right hemithorax was started 4 weeks after the mediastinal irradiation. 20 Gy total mid plane dose was applied in 1.5 Gy fractions. The mediastinal treated volume overlapped to its half width with the left and right hemithorax volumes for increasing the total dose in the mediastinum. Matching problems are clinically negligible at these magnitudes of total dose if applied sequentially to these regions (Fig. 3).

The overall treatment took 23 weeks including the breaks. Complete remission (plain X-ray and CT) lasted for 16 months (Figs 4 and 5).

Local recurrence appeared in the left lumbal area near to the resection site. CT showed a 40 × 75 mm mass involving the end of the resected XIth rib infiltrating the musculature between the left kidney and spleen. An additional 30 Gy was applied and resulted in complete remission of the recurrence.

### Discussion

Metastasising Ewing sarcoma is associated with short term remission and survival but pulmonary irradiation may prove effective.[4–9]

Data are available concerning the prophylactic and therapeutic use of pulmonary irradiation in patients with Ewing sarcoma. *Prophilactically* total doses of 1500–1800 cGy were applied in 150–200 cGy daily fractions within 2.5–3 weeks.[2,10] Doses of the magnitude plus chemotherapy resulted in prolonged disease free survival and prolonged survival in the IESS-I study versus chemotherapy alone.[2] *Therapeutically* the same total doses plus local boost were applied to 2000–2500 cGy[10,11] resulting in complete plus partial remission rates 77%.[11] Pulmonary metastases were inoperable in this young female patient. Primary chemotherapy were associated with severe side effects, although radiation therapy was well tolerated. The left hemithorax and the lower posterior mediastinum were irradiated with the aim of achieving therapeutic effect on manifest multiple lesions. Nevertheless we did the same for prophylactic purposes on the uninvolved right hemithorax. The reasons for the choice of this careful, sequential approach were the following:

- multiple, extensive lesions on the involved left side and therefore palliative aim;
- primary combined treatment was associated with haematological side effects;
- better radiation tolerance of the lung with partial volume-exposure; and
- special importance of the right lung in the respiratory function in case of massive contralateral impairment.

Sequential irradiation of the hemithorax on both

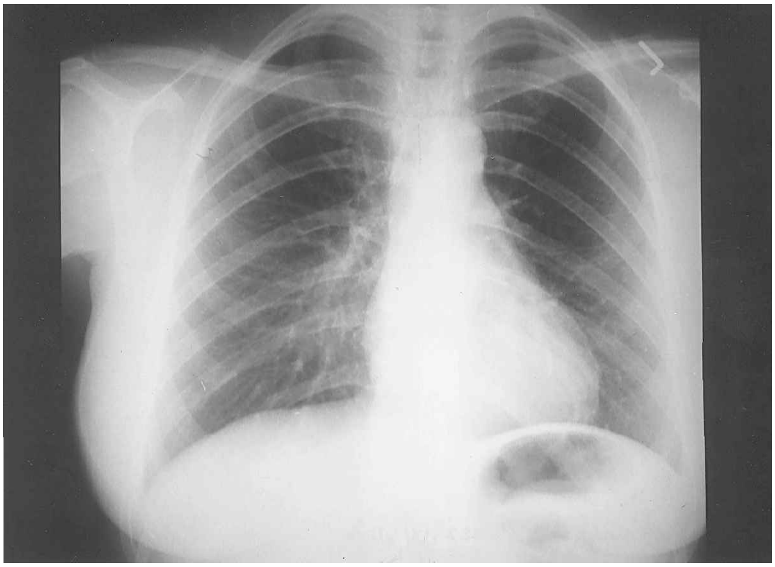

**Fig. 4.** *Plain X-ray 3 months after the finishing irradiation with a total dose of 20 Gy to the left hemithorax.*

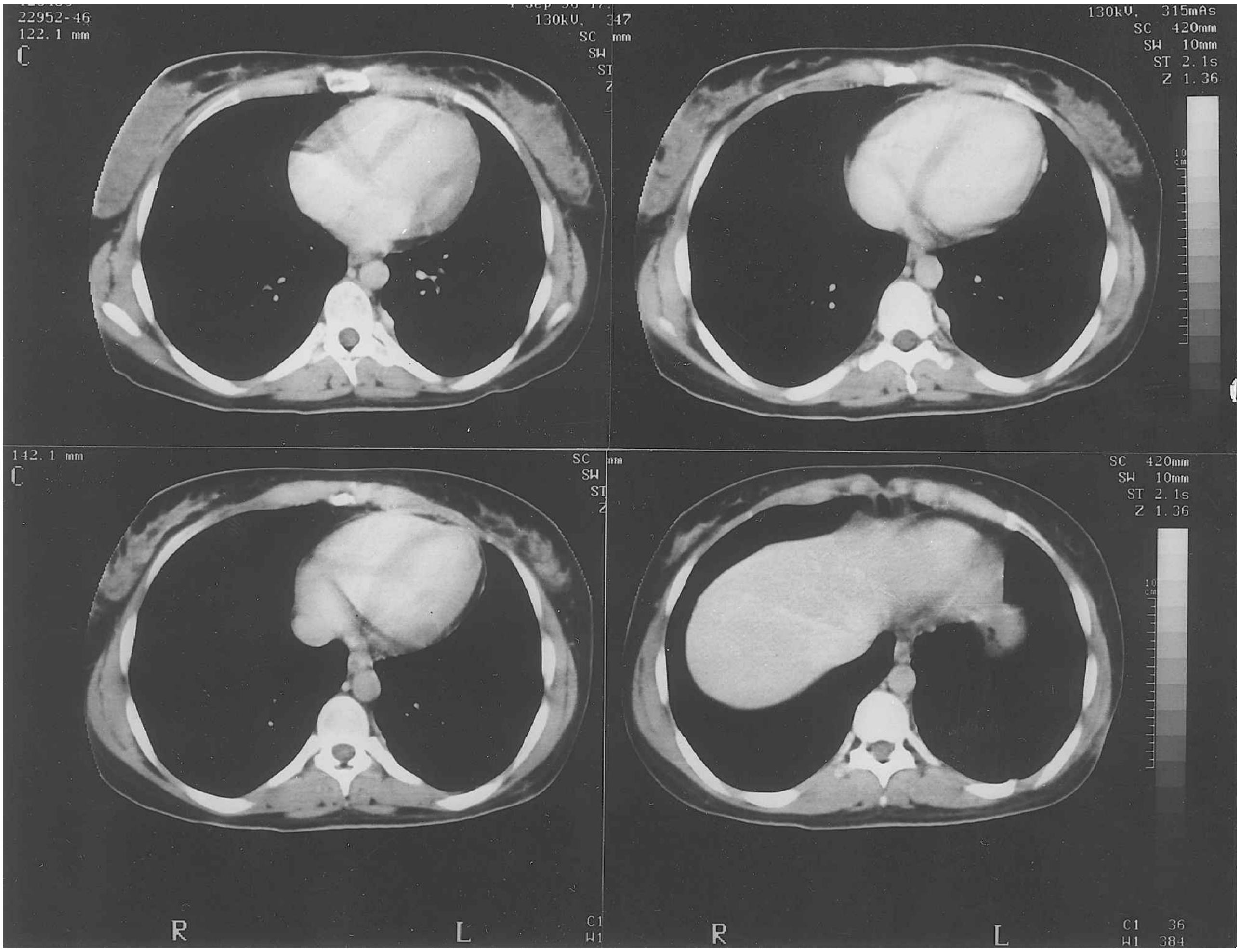

**Fig. 5.** *CT scan demonstrating total remission 3 months after the finishing irradiation with a total dose of 20 Gy to the left hemithorax.*

sides and mediastinum may involve more matching problems than chest irradiation of the two sides at the same time, but the space of time between the treatment phases and the described field arrangement contribute to decrease them at low doses. The outpatient treatment led to radiological complete remission and improvement in quality of life without dyspnoea. She has been symptom free for 16 months after finishing therapy and is able to pursue some sport.

### References

1 Ozaki T, Lindner N, Hoffmann C, Hillmann A, Rodl R, Blasius S, Link T. Ewing's sarcoma of the ribs. A report from the cooperative Ewing's sarcoma study. *European Journal of Cancer* 1995; 31A (13–14):2284–88.

2 Thomas PRM. Ewing's sarcoma. In: Perez CA, Brady LW, eds *Principles and practice of radiation oncology* 2nd edn. Philadelphia: J.B. Lippincott, 1992:1389–95.

3 Roessner A, Mittler U, Rose I, Rading K, Grote H. Pathology of Ewing sarcoma. *Pathologe* 1996; 17(1):6–17.

4 Sandoval C, Meyer WH, Parham DM, Kun LE, Hustu HO, Luo X, Pratt CB. Outcome in 43 children presenting with metastatic Ewing sarcoma: The St. Jude Children's Research Hospital experience, 1962 to 1992. *Medical and Pediatric Oncology* 1996; 26(3):180–5.

5 van Geel AN, Pastorino U, Jauch KW, Judson IR, van Coevorden F, Buesa JM. Surgical treatment of lung metastases: The European Organization for Research and Treatment of Cancer-Soft Tissue and Bone Sarcoma Group study of 255 patients. *Cancer* 1996; 77(4):675–82.

6 Heij HA, Vos A, de Kraker J, Voute PA. Prognostic factors in surgery for pulmonary metastases in children. *Surgery* 1994; 115(6):687–93.

7 Dunst J, Hoffmann C, Ahrens S, Jurgens H. Surgery versus radiotherapy in Ewing's sarcoma with good prognosis. Analysis of the CESS-86 data. *Strahlentherapie und Onkologie* 1996; 172(5):244–8.

8 Dunst J, Jabar S, Paulussen M, Jurgens H. Local therapy of Ewing sarcoma: radiotherapy aspects. *Klinische Pädiatrie* 1994; 206:277–81.

9 Dunst J. The role of radiotherapy in the local treatment of Ewing's sarcoma. *Schweizerische Rundschau Medizinische Praxis* 1995; 84(41):1148–51.

10 Suit RD. Role of therapeutic radiology in cancer of bone. *Cancer* 1975; 35:930–5.

11 Cangir A, *et al.* Ewing's sarcoma metastatic at diagnosis, IESS-MD-I, IESS-MD-II *Cancer* 1990; 66:887–93.
